# AGING ON THE AUTISM SPECTRUM: AN EXPLORATION OF SOCIAL NETWORKS AND IMPLICATIONS

**DOI:** 10.1093/geroni/igz038.2069

**Published:** 2019-11-08

**Authors:** Danielle Waldron, Caitlin Coyle, John Kramer

**Affiliations:** 1 Department of Gerontology, University of Massachusetts Boston, Boston, Massachusetts, United States; 2 Center for Social and Demographic Research on Aging, Gerontology Institute, University of Massachusetts Boston, Boston, Massachusetts, United States; 3 Department of Occupational Therapy, University of Florida, Gainesville, Florida, United States

## Abstract

Social isolation is associated with poor health and well-being in older adults. Little is known about isolation in persons aging on the Autism Spectrum (AS), a group with varied physical and mental health comorbidities. The purpose of this study was to explore social networks of adults aging on the AS. We conducted in-depth interviews (N=30) with adults on the AS (age 50+) and analyzed findings using a constant comparative method. Findings suggest that older adults on the AS struggle to build and maintain social networks over the life course, in large part, because of challenges with communication and trust. Implications of isolation include challenges with community supports and employment. We propose several social convoy models and intervention mechanisms to support this population--as their social networks narrow over time, and they face aging-related challenges without the buffer of strong social relations.

